# Hsp40 co-chaperone Droj2 temporally regulates EcRA dynamics during collective cell migration

**DOI:** 10.3389/fcell.2026.1881376

**Published:** 2026-08-18

**Authors:** Chueh-Wen Wang, Hui-Ying Yu, Kuan-Lin Lai, Nicholas Lee Kong Yau, Yu-Chiuan Chang, Anna C.-C. Jang

**Affiliations:** 1 Department of Biotechnology and Bioindustry Sciences, National Cheng Kung University, Tainan City, Taiwan; 2 Institute of Biomedical Sciences, National Sun Yat-sen University, Kaohsiung, Taiwan

**Keywords:** border cell migration, *Drosophila*, ecdysone receptor (EcR), ecdysone signaling, HSP40 co-chaperones, p160 coactivator, steroid hormone receptor, taiman

## Abstract

Steroid hormone signaling coordinates developmental transitions and tissue morphogenesis, yet how its activity is temporally tuned during collective cell migration remains poorly understood. Here, we identify the Hsp40 co-chaperone Droj2 as a novel regulator of ecdysone signaling during *Drosophila* border cell migration. Through a forward genetic modifier screen, *droj2* emerged as a potent enhancer of Taiman-mediated ecdysone signaling activity. Functional analyses revealed that loss of *droj2* disrupted border cell detachment and directional migration, with earlier depletion causing progressively more severe defects, indicating a critical temporal requirement during migration. Mechanistically, Droj2 depletion selectively triggered precocious downregulation of the nuclear hormone receptor isoform EcRA and premature activation of the ecdysone signaling reporter, *EcRE-lacZ*, during oogenesis, whereas EcRB1 and Taiman expression remained unchanged. In addition to the ovary, Droj2 was also required for proper EcRA nuclear accumulation in larval salivary glands, suggesting a conserved role in coordinating developmental steroid hormone responses across tissues. Together, our findings identify Droj2 as a spatiotemporal regulator of EcRA dynamics that couples chaperone activity to steroid hormone responsiveness during collective migration. This study uncovers a previously unrecognized mechanism linking molecular chaperones with nuclear receptor regulation and developmental timing during tissue morphogenesis.

## Introduction

Collective cell movement is essential for embryogenesis, angiogenesis, wound healing, which relies on the coordinated integration of intrinsic and extrinsic cues that regulate chemotaxis, junctional remodeling, and cytoskeletal dynamics to drive large-scale tissue morphogenesis. Dysregulation of these processes results in developmental abnormalities, impaired wound healing, and cancer metastasis (Mayor and Etienne-Manneville, 2016; Stuelten et al., 2018; Pourjafar and Tiwari, 2024). Importantly, cellular behaviors are governed not only by the presence of signaling inputs but also by their temporal dynamics, as the timing, duration, and sequence of signaling events are critical determinants of biological outcomes (Moss, 2007; Kicheva et al., 2012; Yamanaka et al., 2013; Morrow and Mirth, 2024). However, the molecular mechanisms that coordinate and integrate these temporal cues to ensure orderly execution of complex cellular behaviors remain poorly understood.

*Drosophila* border cell migration provides a well-established *in vivo* model for investigating the spatiotemporal control of collective cell movement (Montell, 2003; Montell et al., 2012; Rorth, 2012). At stage 9 of oogenesis, 6-8 anterior follicle cells are specified and recruited by JAK/STAT signaling to form a migratory cluster (Silver and Montell, 2001). Notably, JAK/STAT signaling also functions early in oogenesis to regulate follicle cell fate determination, highlighting the importance of temporal regulation in its activity. The bHLH domain protein Dysfusion (Dysf) acts as a temporal switch that promotes robust nuclear accumulation of STAT, thereby maximizing pathway activity to ensure precise spatiotemporal specification of border cell fate (Wu et al., 2022). Coordinated with overall oogenesis, border cell detachment at stage 9 from the anterior follicular epithelium and subsequent collective migration toward the oocyte are scheduled by the steroid hormone signaling (Bai et al., 2000; Jang et al., 2009).

The steroid hormone 20-hydroxyecdysone (ecdysone) functions as a central endocrine signal regulating key developmental transitions, including larval molting, neuronal remodeling, tissue reorganization during metamorphosis, and adult gonad development (Thummel, 1996; Yamanaka et al., 2013; Belles and Piulachs, 2015). Ecdysone signals through a nuclear receptor complex composed of the ecdysone receptor and Ultraspiracle (EcR/Usp), which recruits the *Drosophila* p160 coactivator Taiman (Tai) to activate transcription of downstream target genes (Koelle et al., 1991; Riddiford, 1993; Yao et al., 1993; Bai et al., 2000). Accordingly, disruption of any component within this signaling axis impairs oogenesis and inhibits border cell migration. Tai is evolutionarily conserved as a member of the SRC family of coactivators that regulate nuclear receptor-dependent gene expression (Xu and Li, 2003). Notably, dysregulation of SRC proteins has been implicated in multiple human diseases, particularly hormone-dependent cancers (Anzick et al., 1997; Lonard and O'Malley, 2005). Collectively, these findings highlight the conserved role of ecdysone signaling in coordinating developmental timing and underscore its broader relevance to hormone-dependent disease processes.

Reporter activity of *EcRE-lacZ* (Kozlova and Thummel, 2002) reflects ecdysone signaling dynamics, which gradually increase from stage 9 and peak at stage 10, coinciding with the timing of border cell detachment. This signaling output is modulated by the bHLH domain of Tai through its interaction with the transcriptional repressor Abrupt (Ab). Expression of a bHLH-deleted form, Tai(ΔB), in border cells results in hyperactivation of ecdysone signaling and precocious migration accompanied by elevated JAK activity. Consistently, progressive downregulation of Ab relieves repression on Tai, enabling stepwise activation of target genes during migration (Jang et al., 2009). In addition to coactivator regulation, ecdysone signaling is further tuned by distinct isoforms of the ecdysone receptor. EcR exists as two major isoforms, EcRA and EcRB1, which share conserved DNA binding, ligand-binding and AF-2 domains but differ in their N-terminal AF-1 regions. These isoforms exert differential effects on ecdysone-responsive transcription during developmental transitions and oogenesis (Cherbas et al., 2003; Jang et al., 2009). However, the mechanisms governing EcR activity and expression remain poorly defined. Addressing this gap is essential not only for understanding temporal control of border cell migration and developmental transitions, but also for elucidating general principles underlying hormone receptor-dependent disease progression.

To elucidate the regulation of ecdysone signaling, we performed a genetic screen and identified the *Drosophila* Hsp40 chaperone Droj2 as a previously unrecognized regulator of border cell migration and a potent enhancer of ecdysone signaling activity. Functional analyses demonstrate that Droj2 is intrinsically required for border cell motility, as both loss- and gain-of-function perturbations impair migration. Mechanistically, depletion of droj2 induces precocious downregulation of EcRA, a key negative regulator of ecdysone signaling during oogenesis (Jang et al., 2009), leading to premature activation of *EcRE-lacZ* as early as stage 8. A similar suppression of EcRA nuclear accumulation was observed in larval salivary glands during larva development, indicating a conserved role for Droj2 in regulating steroid hormone signaling. Collectively, these findings establish Droj2 as a temporal modulator of ecdysone signaling and highlight its spatiotemporal control of EcRA expression during *Drosophila* development.

## Results

### Droj2 is identified as a novel regulator to modulate ecdysone signaling in *Drosophila* border cell migration

During *Drosophila* oogenesis, border cells are recruited by anterior polar cells to form a migratory cohort (Supplementary Figure S1A). The detachment of this cluster from the anterior follicular epithelium at stage 9 is temporally regulated by a gradual increase in ecdysone signaling (Jang et al., 2009). Ecdysone signaling is mediated by the hormone receptor complex EcR/Usp, which recruits the p160 coactivator Taiman (Tai) to the general transcriptional machinery in a ligand-dependent manner, (Bai et al., 2000). The activity level of this pathway is modulated by the bHLH domain of Taiman through its interaction with the transcriptional repressor Abrupt. Accordingly, deletion of the bHLH domain (Tai(ΔB)) results in hyperactivation of ecdysone signaling, leading to precocious border cell migration in co-expression of hyperactive JAK at stage 8 (Jang et al., 2009). To identify novel regulators of steroid hormone signaling that temporally control border cell migration, we performed a forward genetic screen for modifiers of Tai(ΔB)-dependent migration defects. For this screen, *UAS-tai(ΔB)23A4* was specifically expressed in border cells using the GAL4-UAS system (Brand and Perrimon, 1993) under the control of the *slbo* promoter (Rørth et al., 1998) (Supplementary Figure S1BA).

**FIGURE 1:**
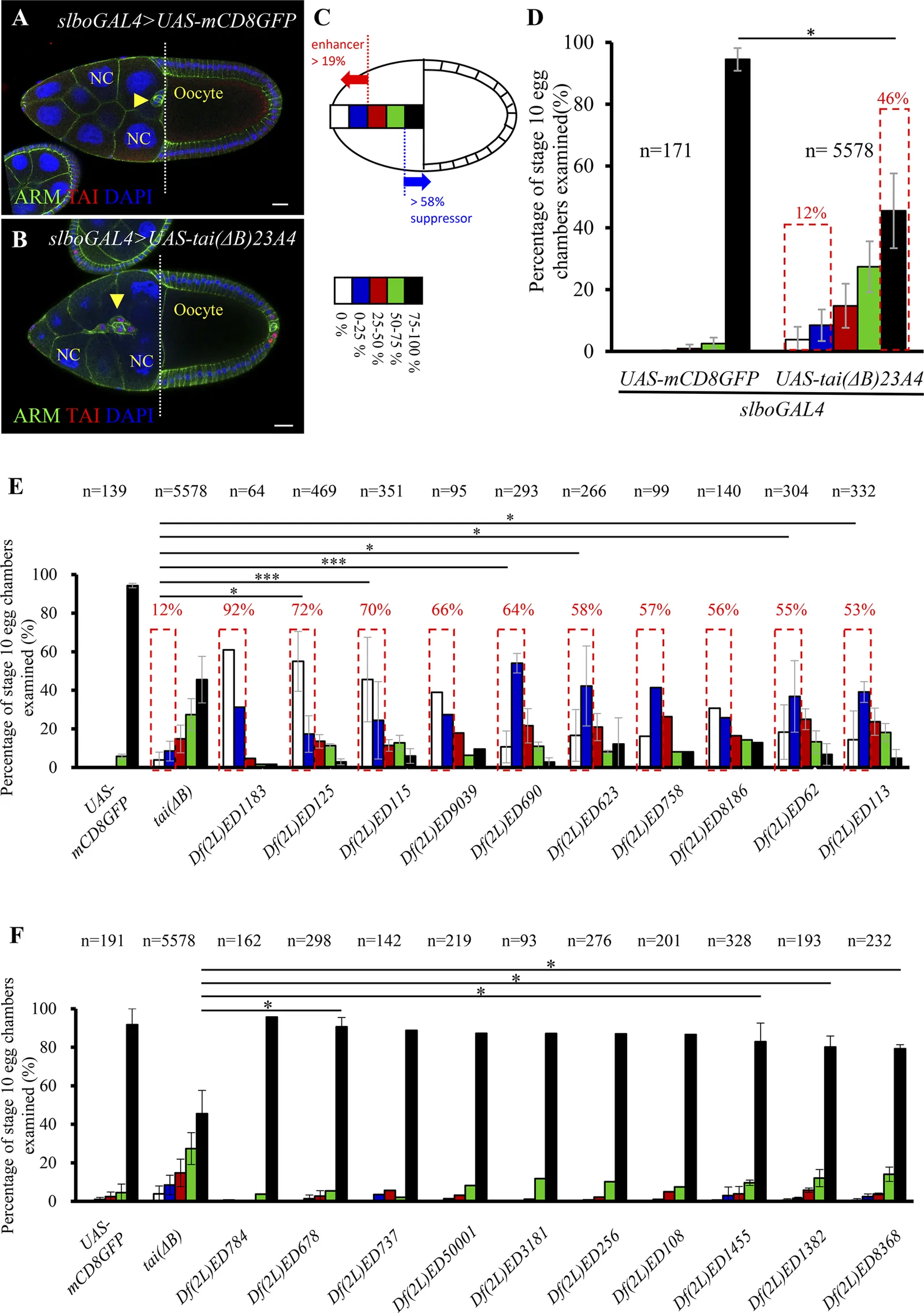
A genetic screen for deficiency lines modifying Tai(ΔB) induced border cell migration defects. All UAS transgenes were expressed under the control of *slboGAL4* **(A,B)** Confocal images showing border cells (yellow arrowheads) migrate through the nurse cells (NC) toward the oocyte boundary (white dashed line). The egg chambers are stained with anti-Armadillo (green), DAPI (blue) to mark nuclei, and anti-Taiman (red) to validate transgene expression. Compared with the control expressing *UAS-mCD8GFP* **(A)**, expression of *UAS-tai(ΔB)23A4* **(B)** results in migration defects **(C)** Schematic illustration of migration index for quantitative assessment. Border cell migration was divided into five sections based on the relative distance traveled toward the oocyte border: 0% (no detachment; white), 0%–25% (blue), 25%–50% (red), 50%–75% (green), and 75%–100% (complete migration; black). Screening criteria used to define deficiency lines as enhancers or suppressors of *UAS- tai(ΔB)*-mediated migration defects **(D–F)** Quantification of border cell migration for the indicated genotypes. Quantitative analysis of modifier effects in the *UAS-tai(ΔB)23A4* background revealed the top 10 candidates that significantly enhance **(E)** or suppress **(F)** the migration defect. **(E,F)** Data are presented as mean ± SD (standard deviation). Statistical significance was determined using an unpaired two-tailed Student’s t-test. *P < 0.05; **P < 0.01; ***P < 0.001. Scale bars: 20 μm. N indicates the number of stage 10 egg chambers examined.

Border cells expressing *UAS-tai(ΔB)23A4* (Figure 1B, yellow arrowheads) exhibited a pronounced migration defect, with only 46% of clusters reaching the oocyte boundary, while the remainder stalled along the migration path (Figures 1B,D). In contrast, in control egg chambers, 95% of border cells expressing UAS-mCD8GFP successfully completed migration by stage 10 (Figure 1A, yellow arrowheads). To quantitatively assess migration, the distance from the anterior epithelium to the oocyte boundary was divided into five categories: 0%, 0%–25%, 25%–50%, 50%–75%, and 75%–100%, represented by white, blue, red, green, and black, respectively (Figure 1C). In our screen, analysis of 5,578 stage 10 egg chambers expressing UAS-*tai(ΔB)23A4* revealed a pronounced border cell migration defect. Whereas 95% of border cell clusters in the control (*UAS-mCD8GFP*) completed migration, only 46% of Tai(ΔB)-expressing clusters reached the oocyte, representing a 49% reduction in migration efficiency (Figure 1D). To identify genetic modifiers of the Tai(ΔB)-induced migration phenotype, we crossed this line with *Drosophila* melanogaster Df (2L)ED and Df (2R)ED deficiency lines carrying deletions on chromosome II(Ryder et al., 2007). F1 female progeny were analyzed for border cell morphology and migration defects (Supplementary Figure S2A). The selection criteria of genetic enhancers or suppressors were established based on the baseline motility defect induced by *UAS-tai(ΔB)*. In this background, 12% of examined stage 10 egg chambers exhibited severe migration defects, falling within the 0% and 0%–25% migration categories, with a standard deviation of 7%. Accordingly, any deficiency line in which more than 19% of border cell clusters stalled within the 0%–25% range was classified as a candidate enhancer (Figure 1C; Supplementary Figure S2B). Similarly, only 46% ± 12% of border cell clusters expressing *UAS-tai(ΔB)* successfully completed migration to the oocyte boundary. Based on these criteria, deficiency lines were classified as candidate suppressors when more than 58% of clusters reached the oocyte border (Figures 1C,D; Supplementary Figure S2B). A total of 233 deficiency lines spanning chromosome II (green regions in Supplementary Figure S2B) were screened, leading to the identification of 78 enhancer and 38 suppressor candidates. The top 10 enhancer candidates (red regions in Supplementary Figure S2B) and suppressor candidates (blue regions in Supplementary Figure S2B) were selected for further validation. Among these, only deficiency lines that showed statistically significant enhancement (Figure 1E) or suppression (Figure 1F) of the migration phenotype were prioritized for subsequent analyses. Statistical comparisons were not performed for those genotypes (Figures 1E,F) because their lethality prevented us from obtaining the minimum of three independent biological replicates required for statistical analysis.

**FIGURE 2:**
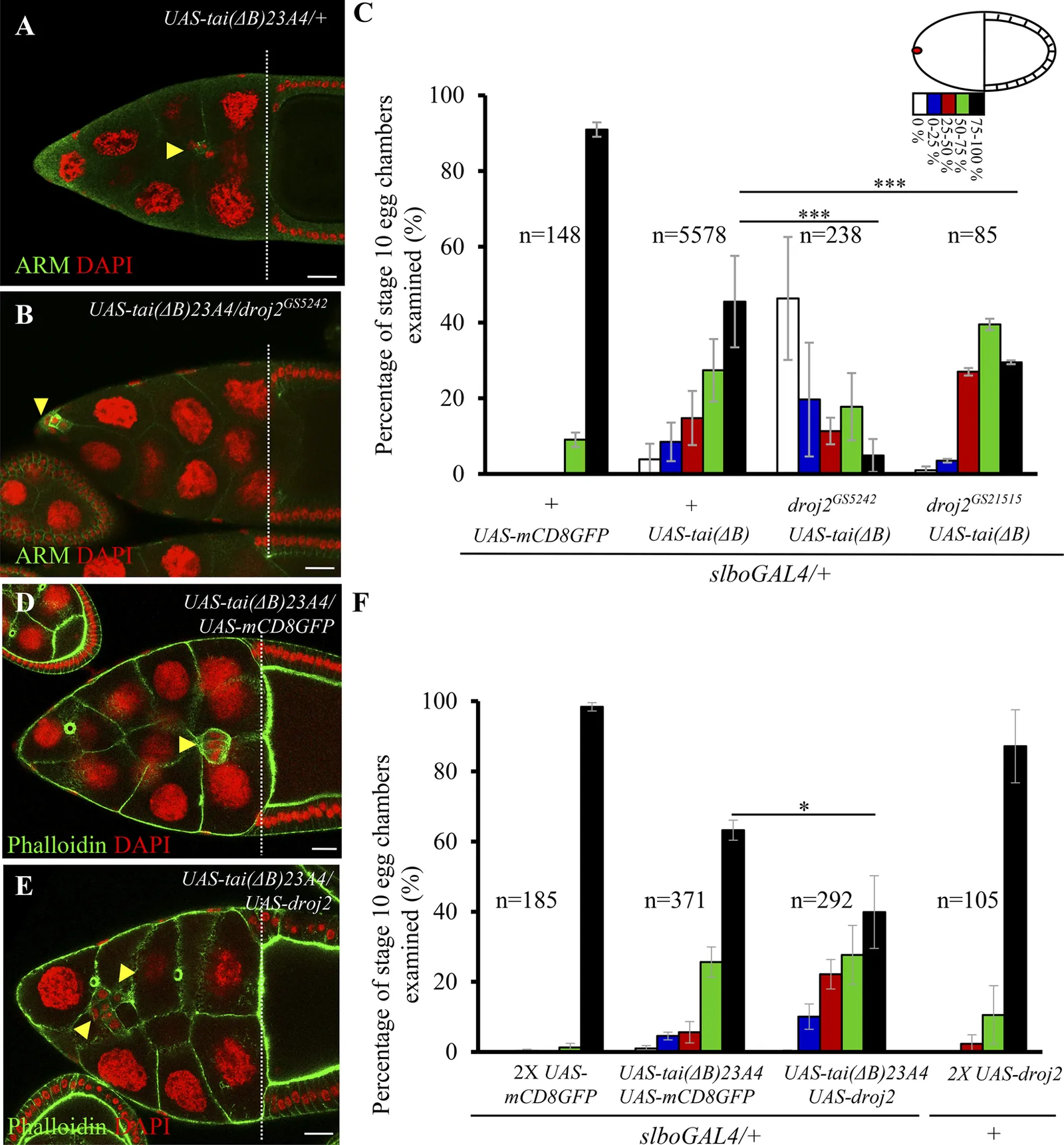
Dysregulation of *droj2* enhances border cell migration defects caused by Tai(ΔB) **(A,B,D,E)** Representative confocal images of stage 10 egg chambers. Nuclei are labeled with DAPI (red). Cell morphology is visualized by anti-Armadillo (green, **(A,B)** or phalloidin (green, **(D,E) (D,E)**. Migrating border cell clusters are indicated by yellow arrowheads. Both heterozygous *droj2* mutant alleles **(A–C)** and *UAS-droj2* overexpression **(D–F)** enhance migration defects induced by *UAS-tai(ΔB)*. Data are presented as mean ± SD. Statistical significance was assessed using an unpaired two-tailed Student’s t-test. *P < 0.05; **P < 0.01; ***P < 0.001. Scale bars, 20 μm. N indicates the number of stage 10 egg chambers analyzed.

Among the top 10 enhancer or suppressor candidates, a total of 893 genes were mapped within these genomic intervals (Supplementary Figure S2B, C). To further refine candidate selection, this gene set was cross-referenced with datasets from two previously published screens related to ecdysone signaling (Domanitskaya et al., 2014; Ables et al., 2016) respectively, yielding 25 candidates. We further requested mutants or RNAi lines to validate their phenotypes in border cells. Among these, 20 candidates exhibited significant suppression, while four mutants were identified as enhancers (Supplementary Figure S3A). Several mutants exhibited strong suppression of the Tai(ΔB)-induced migration defect, including *lgl* (*lethal giant larvae*, 99%), *spin* (*spinster*, 98%), and *trs23* (*transport protein particle 23*, 95%), which encode proteins involved in cell polarity, lysosomal trafficking, and ER-to-Golgi vesicle transport, respectively. Notably, the *droj2* ^*GS5242*^ insertion line at the *droj2* locus, which encodes a *Drosophila* type I Hsp40 chaperone, exhibited a severe migration defect, with only 5% of border cell clusters completing normal migration (Supplementary Figure S3A). Another allele, *droj2* ^*GS21515*^, similarly enhanced the Tai(ΔB)-induced migration defect with only 30% of examined egg chambers completing migration (Figures 2A–C; Supplementary Figure S2B). To further validate the functions of candidate genes retrieved from the deficiency screen, available mutants and RNAi lines were analyzed for their effects on border cell migration using either the *slboGAL4* or *c306GAL4* drivers (Supplementary Figure S2B, 3B,C) (Barth et al., 2012). Whereas *slboGAL4* induces transgene expression after stage 9 of oogenesis (Supplementary Figure S1B), *c306GAL4* is activated much earlier, beginning in the germarium (Supplementary Figure S1C). Knockdown of *droj2* alone using either driver significantly impaired border cell migration (Supplementary Figure S3B,C), indicating that Droj2 is required throughout multiple stages of border cell migration. In agreement, gain-of-function of Droj2 also exacerbated migration defects (Figures 2D–F). These results indicate that *droj2* genetically interacts with Tai(ΔB), and that its activity is crucial for proper collective cell migration in *Drosophila*. We therefore sought to further investigate the role of *droj2* in *Drosophila* oogenesis.

**FIGURE 3:**
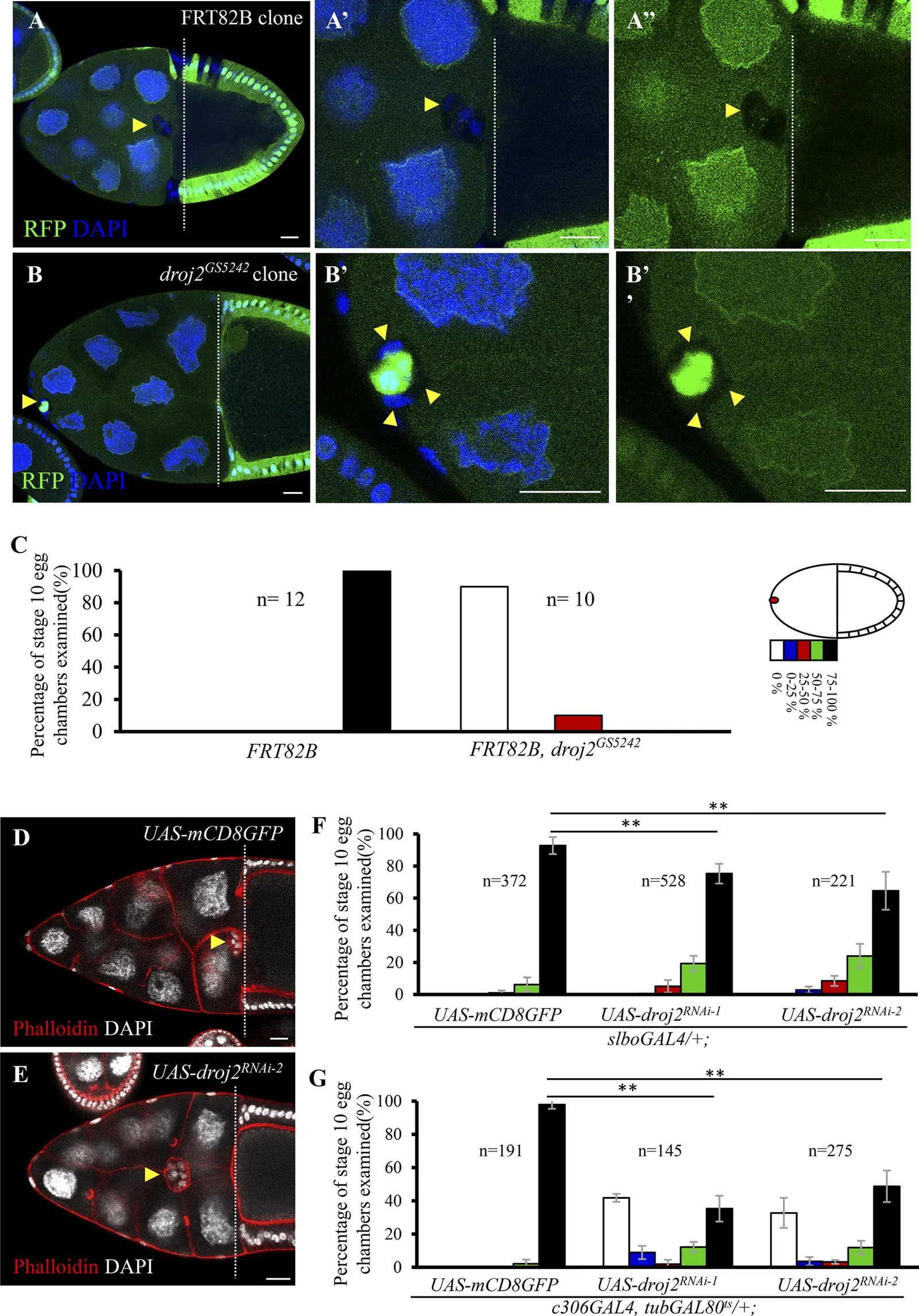
*droj2* is required for border cell migration (A-B″) Mosaic clones were generated using the FLP/FRT system at the FRT82B locus. Mutant clones are identified by loss of RFP (yellow arrowheads; green represents RFP). Nuclei are labeled with DAPI (blue). Higher-magnification views of **(A,B)** are shown in **(A′-A″)** and **(B′-B″)**, respectively. **(A)** Control FRT82B clones exhibit normal border cell migration. **(B)** Homozygous *droj2* ^*GS5242*^ mutant clones in border cells (yellow arrowheads) display migration failure (white dashed line). **(C)** Quantification of migration defects for the indicated genotypes. **(D,E)** Representative confocal images of stage 10 egg chambers showing border cell migration (yellow arrowheads). Nuclei are labeled with DAPI (white), and F-actin is visualized by phalloidin staining (red) to outline cell morphology. **(F,G)** RNAi knockdown of *droj2* driven by *slboGAL4* or *c306GAL4* significantly impairs migration compared with the control (*UAS-mCD8GFP*). Data are presented as mean ± SD. Statistical significance was determined using an unpaired two-tailed Student’s t-test. *P < 0.05; **P < 0.01; ***P < 0.001. Scale bars, 20 μm. N indicates the number of stage 10 egg chambers investigated.

*droj2* encodes a type I Hsp40/DnaJ family co-chaperone that regulates Hsp70-dependent protein folding and stability and has been implicated in steroid hormone receptor binding and turnover (Wang and Brock, 2003; Gaestel, 2006). Thus, we first examined whether Droj2 is required for *Drosophila* ovarian development, border cell migration, or metamorphosis, processes that are all regulated by ecdysone signaling. To specifically assess its role in border cell migration, we generated homozygous clones of the *droj2* ^*GS5242*^ allele in border cells using the FLP/FRT system (Xu and Rubin, 1993). In control clones marked by the absence of Red Fluorescence Protein RFP (RFP; yellow arrowheads, Figure 3A,A’), border cell clusters migrated normally and reached the oocyte border by stage 10. In contrast, *droj2* ^*GS5242*^ mutant clones in border cells exhibited severe migration defects (Figure 3B,B’), with 90% of clusters stalling at the anterior tip of the egg chamber (Figure 3C). By comparison, 100% of control clones completed migration (Figures 3A,C).

To further assess the requirement of *droj2*, we knocked down its expression using RNA interference driven by *slboGAL4* (Figures 3D–F). Compared with controls (Figure 3D), *UAS-droj2* ^RNAi^ caused mild migration defects, with 25%–35% of clusters failing to reach the oocyte border in stage 10 (Figures 3E,F). Notably, earlier knockdown of *droj2* driven by *c306GAL4* caused more severe defects, with 51%–65% of border cell clusters failing to complete migration and 33%–42% remaining attached to the anterior epithelium (Figure 3G). These findings indicate that *droj2* function is particularly critical prior to stage 9 of oogenesis, specifically during border cell detachment, rather than during the later phases of migration.

We next tested whether *droj2* overexpression alone is sufficient to disrupt border cell migration by inducing *UAS-droj2* expression using *slboGAL4*. In control egg chambers expressing *UAS-mCD8GFP*, border cell clusters migrated normally and reached the oocyte border (Supplementary Figure S4A,D). In contrast, overexpression of Droj2 using two independent *UAS-droj2* insertion lines resulted in migration defects in 28%–30% of clusters (Supplementary Figure S4B-D). Together, our results demonstrated that perturbation of Droj2 expression, either by depletion or ectopic overexpression, disrupts normal border cell migration.

**FIGURE 4:**
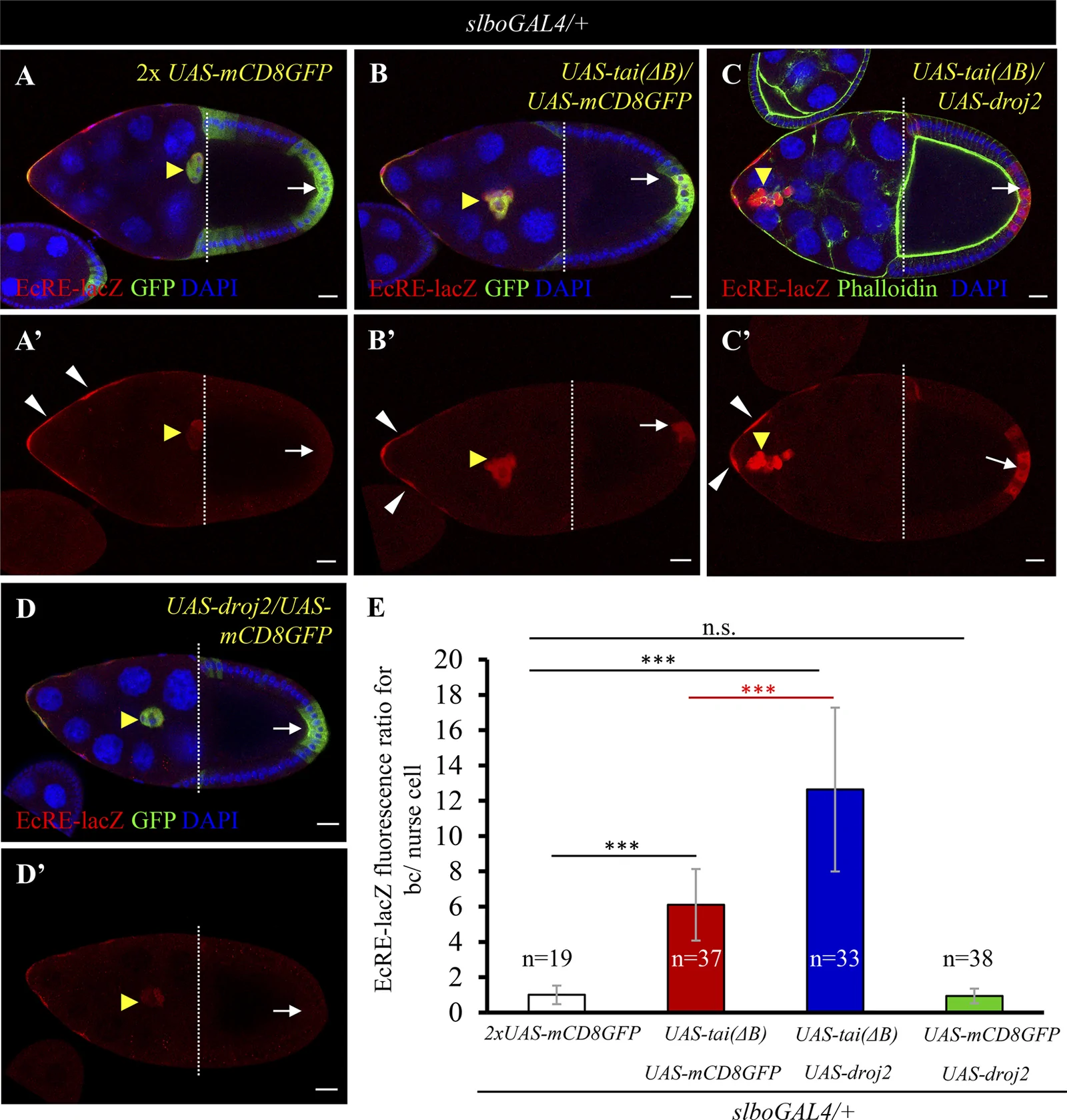
Overexpression of *droj2* enhances Tai(ΔB)-mediated hyperactivation of ecdysone signaling. **(A–D)** *EcRE-lacZ* activity is indicated by anti-βgal staining (red). GFP marks the transgene expression domain induced by *slboGAL4*, and DAPI labels nuclei to outline egg chamber morphology. **(E)** Quantification of *EcRE-lacZ* activity shown as the fluorescence intensity ratio of border cells (bc) to nurse cells (nc) for the indicated genotypes. Stretching follicle cells (white arrowheads), posterior follicle cells (white arrows), and border cells (yellow arrowheads) are indicated. Data are presented as mean ± SD. Statistical significance was determined using an unpaired two-tailed Student’s t-test. n. s., not significant; *P < 0.05; **P < 0.01; ***P < 0.001. Scale bars, 20 μm. N indicates the number of stage 10 egg chambers investigated.

This screen was designed to identify novel loci that regulate ecdysone signaling, and *droj2* was identified as a strong enhancer of Tai(ΔB)-dependent motility defects (Figures 2A–C). To determine whether ecdysone signaling is altered under dysregulation of *droj2*, we examined the activity of the *EcRE-lacZ* reporter (Kozlova and Thummel, 2002). Anti-βgal staining revealed robust activation of ecdysone signaling in anterior follicle cells, including stretching cells (white arrowheads) and border cells (yellow arrowheads; Figures 4A,A’). Overexpression of *UAS-tai(ΔB)* led to a 6-fold increase in *EcRE-lacZ* expression (Figures 4B,B’,E) and induced ectopic activation in posterior follicle cells (white arrows; Figures 4B,B’). Notably, co-expression of a single copy of *UAS-droj2* further elevated *EcRE-lacZ* activity to approximately 13-fold (Figures 4C,C’,E), indicating that Droj2 enhances ecdysone signaling activity. However, expression of Droj2 alone did not alter *EcRE-lacZ* activity (Figures 4D,D,’,E). Collectively, these results suggest that Droj2, a *Drosophila* Hsp40 chaperone, is required for border cell migration by modulating Tai-dependent ecdysone signaling.

**FIGURE 5:**
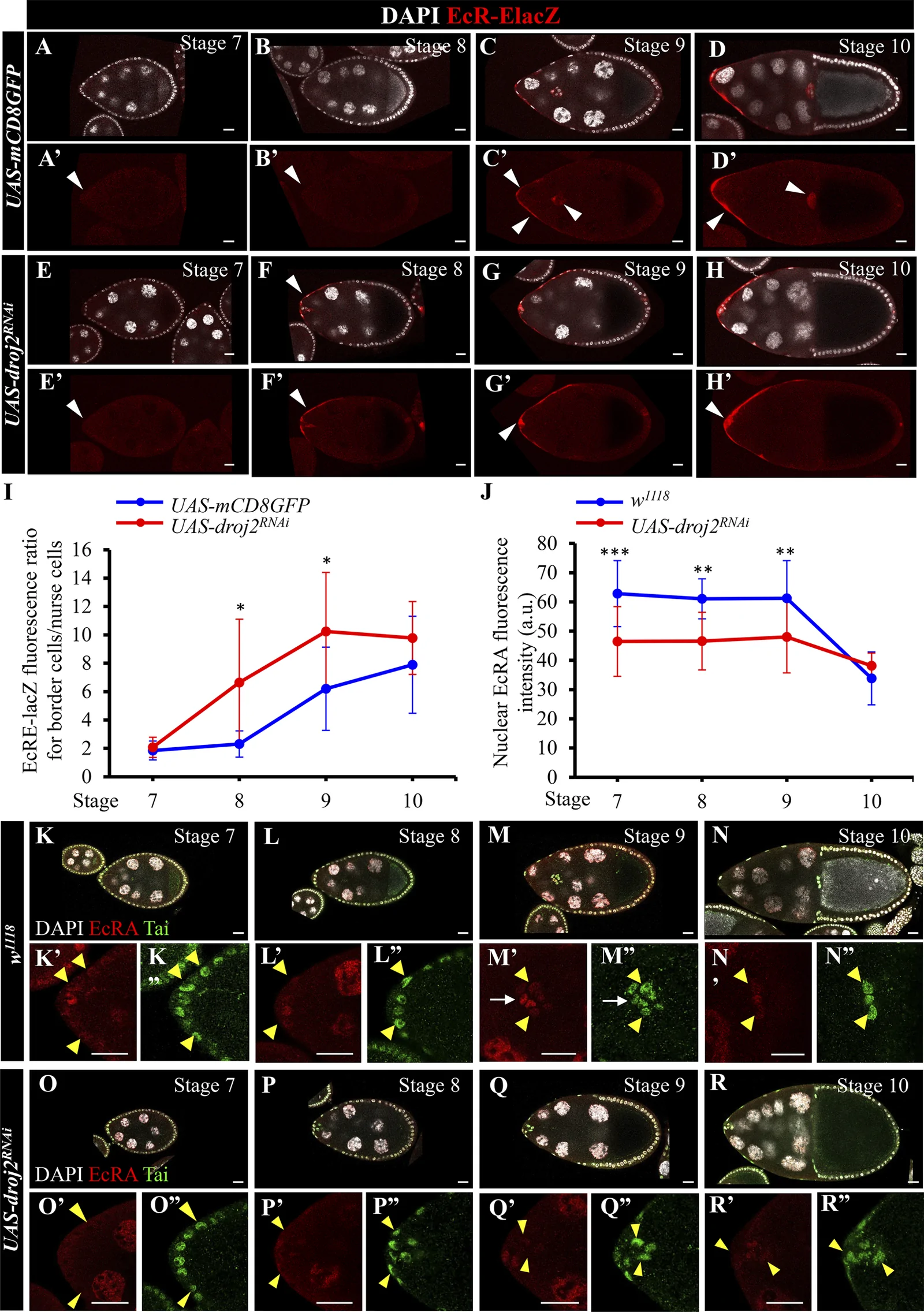
Loss of *droj2* results in precocious activation of the ecdysone signaling. DAPI (white) marks nuclei in all egg chambers (A-H, K-R). **(A–D)** In *UAS-mCD8GFP* expressed egg chambers, *EcRE-lacZ* activity, detected by anti-β-gal staining (white arrowheads, red), is low or undetectable at stages 7–8 **(A,B′)**, increases at stage 9 **(C-C′)**, and reaches peak levels at stage 10 **(D-D′)**. **(E–H)** Expression of *UAS-droj2* ^*RNAi*^ by *c306GAL4* induces precocious activation of *EcRE-lacZ*, with detectable signal already at stage 8 **(F-F′)**. **(I)** A total of 129 follicle cells were analyzed to quantify *EcRE-lacZ* activity across the indicated genotypes and stages. **(J)** Quantification of EcRA fluorescence intensity from 155 follicle cells for the indicated genotypes and stages. **(K–N)** In *w* ^*1118*^ egg chambers that served as the control, EcRA expression is progressively downregulated in the nuclei of anterior follicle cells from stage 7 onward (yellow arrowheads), while its expression is retained in polar cells (white arrows, **(M′ and M″)**). **(O–R)** Knockdown of *droj2* induced by *c306GAL4* further accelerates the downregulation of EcRA. Anti-Tai staining (green, K-R) serves as a control to visualize egg chamber nuclear morphology. Statistical significance was determined using an unpaired two-tailed Student’s t-test. *P < 0.05; **P < 0.01; ***P < 0.001. Scale bars, 20 μm.

Hsp40 proteins recognize unfolded or nascent polypeptides and deliver them to Hsp70, promoting client folding and stabilization, often followed by transfer to Hsp90 for final maturation (Echeverria and Picard, 2010; Kampinga and Craig, 2010). A well-characterized example is the glucocorticoid receptor (GR), whose ligand-responsive state is maintained by the Hsp40-Hsp70-Hsp90 chaperone machinery. Upon hormone binding, GR is stabilized, enabling its nuclear translocation and transcriptional activation (Beato, 1989; Htun et al., 1996). In contrast, estrogen receptor (ER) and *Drosophila* ecdysone receptor (EcR) exhibit predominant nuclear localization and ligand-independent properties (Truman et al., 1994; Bai et al., 2000; Carney and Bender, 2000; Stenoien et al., 2000; Stenoien et al., 2001; Maruvada et al., 2003). Based on these observations, Droj2 may influence the functional state of EcR, thereby enhancing Tai(ΔB)-dependent signaling without independently activating *EcRE-lacZ*. To test this hypothesis, we examined the expression of the coactivator Taiman (Tai) and the ecdysone receptor isoforms EcRA and EcRB1 in Droj2 depletion induction by *c306GAL4* and assessed their correlation with *EcRE-lacZ* activity across different stages of oogenesis. Egg chamber stages were determined based on the anterior-posterior to dorsal-ventral (AP/DV) axis ratio (Alegot et al., 2018), which progressively increases from〜1.6 to〜2.1 between stages 7 and 10 (Supplementary Figure S5J). Importantly, knockdown of *droj2* led to precocious activation of *EcRE-lacZ* in border cell precursors as early as stage 8 (white arrowheads; Figures 5F,F’), a phenotype not observed in wild-type egg chambers, where reporter activity is minimal at stages 7–8 (Figures 5A,B) and only becomes evident at early stage 9 and peaks at stage 10 (Figures 5C,D). Quantitative analysis confirmed a significant premature induction of *EcRE-lacZ* at stage 8 upon *droj2* knockdown (Figure 5F,F’,I), reaching levels comparable to those observed in wild-type stage 9 egg chambers (Figures 5C,C’,I), coinciding with the onset of border cell detachment. Furthermore, *EcRE-lacZ* expression increased ∼10-fold at stage 9 in *UAS-droj2* ^*RNAi*^ *-*expressing cells, reaching peak levels that were sustained through stage 10 (Figures 5G–I).

Ecdysone receptors and the co-activator, Tai, are expressed uniformly throughout oogenesis (Bai et al., 2000). However, a gradual downregulation of EcRA accompanied by enrichment of EcRB1 in anterior follicle cells has been implicated in the transition from a repressor-dominant state (EcRA) to an activator-dominant state driven by EcRB1 upregulation during stage 9 (Jang et al., 2009). From stages 7–10 of oogenesis, we observed a progressive downregulation of EcRA (Figures 5J–N), while EcRB1 and Tai remained relatively constant (Supplementary Figure S5A-D, I). However, upon *UAS-droj2* ^*RNAi*^ expression, the nuclear intensity of EcRA in stage 7–8 anterior follicle cells (Figures 5J,O’,P’) was markedly reduced to levels in comparison to those observed in wild-type late stage 9–10 border cells (Figures 5J,M’,N’). This reduced EcRA level was maintained in Droj2-depleted border cells (yellow arrowheads; Figure 5Q’,R’), similar to the low EcRA expression observed in wild-type border cells at these stages (yellow arrowheads; Figure 5M’,N’). Interestingly, the precocious downregulation of EcRA upon *droj2* knockdown was specific, as EcRB1 staining (Supplementary Figure S5E-H) and Tai expression (Figures 5O–R; Supplementary Figure S5E-H) remained unchanged compared with wild type (Figures 5K–N; Supplementary Figure S5A-D).

**FIGURE 6:**
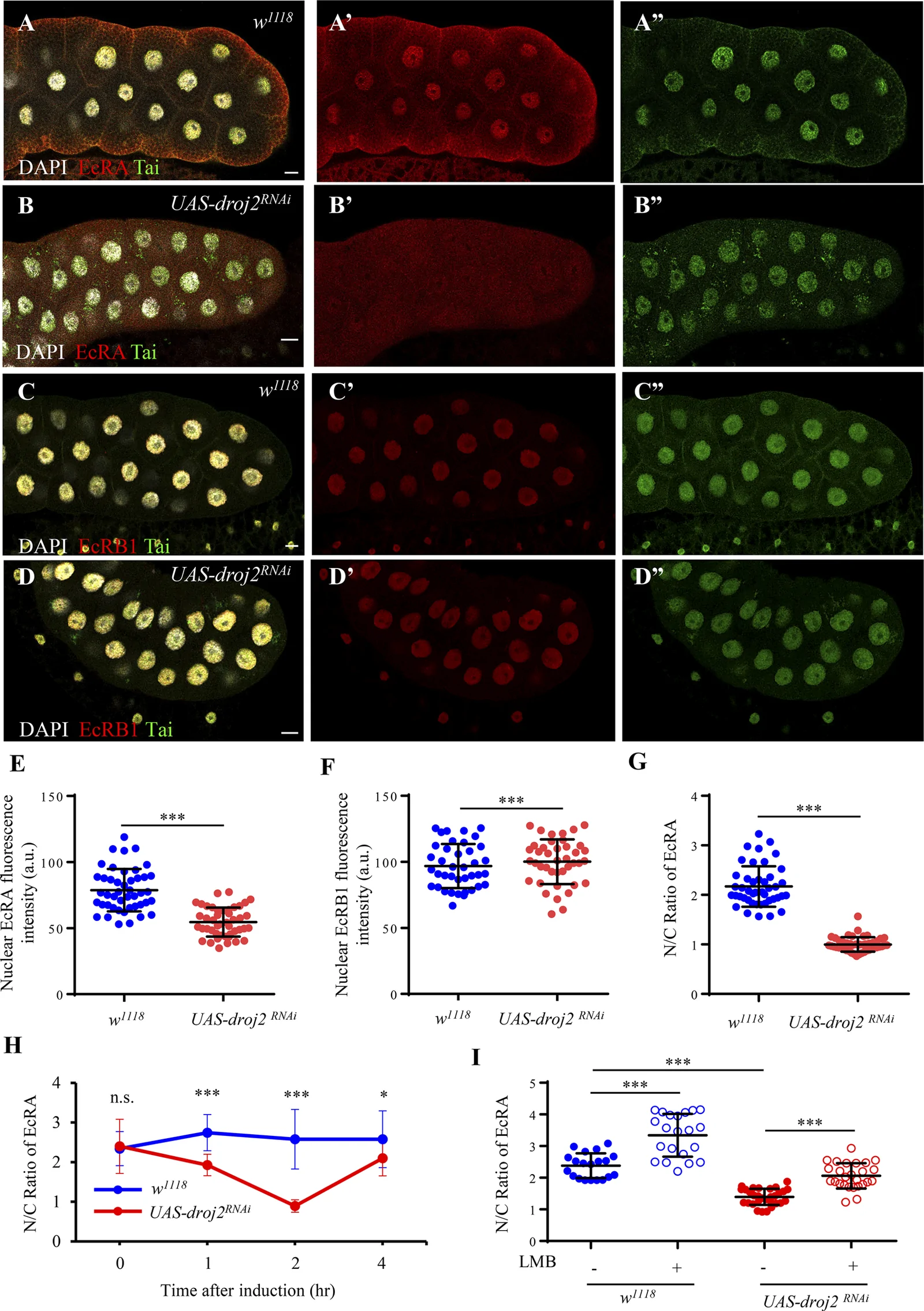
Droj2 suppression alters nuclear EcRA expression in *Drosophila* salivary gland. Nuclear EcRA (anti-EcRA, red) is prominently detected in *w* ^*1118*^ salivary glands **(A,A′)** but is markedly reduced upon *UAS-droj2* ^*RNAi*^ expression induced by *24BGAL4* **(B,B′)**. In contrast, the levels of EcRB1 (red; **(C, C′, D,D’)**) and Taiman (anti-Tai, green) remain unchanged under these conditions. **(E)** Quantitative assessment for nuclear fluorescent intensity of EcRA in 91 salivary gland cells. **(F)** Quantification of nuclear EcRB1 intensity in total 80 salivary gland cells. **(G)** Quantification of the EcRA nuclear-to-cytoplasmic (N/C) ratio in salivary gland cells (n = 90 cells for all indicated genotypes). **(H)** Time-course analysis of the EcRA N/C ratio in the control (*w* ^*1118*^) and *UAS-droj2RNAi* salivary gland cells (n = 183 cells for all indicated genotypes). **(I)** Quantification of the EcRA N/C ratio in the indicated genotypes following treatment with (+LMB) or without (−LMB) Leptomycin B (LMB) (n = 97 cells for all indicated genotypes). Statistical significance was determined using an unpaired two-tailed Student’s t-test. n. s., not significant; *P < 0.05; **P < 0.01; ***P < 0.001. Scale bars, 20 μm.

We next examined whether Droj2 similarly regulates EcRA in other ecdysone-mediated developmental contexts. During *Drosophila* larval salivary gland development, ecdysone signaling governs secretory protein biosynthesis, secretion, polytene chromosome puffing, gene activation, and ultimately programmed cell death during metamorphosis (Walker and Ashburner, 1981; Thummel, 1996; Biyasheva et al., 2001; Thummel, 2001; Uyehara and McKay, 2019). Consistent with our observations in the ovary, Droj2 downregulation by *24BGAL4* led to a reduction in nuclear EcRA expression in salivary glands (Figure 6B,B’), a phenotype not observed in the wild type (Figure 6A). In contrast, Tai (Figures 6A–D) and EcRB1 expression (Figures 6C,C’,D,D’) remained unchanged upon *droj2* knockdown. Quantitative analysis further showed that nuclear EcRA intensity decreased from 79 to 55 arbitrary units (a.u.) in *UAS-droj2* ^*RNAi*^ expression (Figure 6E), whereas EcRB1 levels were not significantly affected (Figure 6F). Interestingly, the nuclear-to-cytoplasmic (N/C) ratio of EcRA was approximately 2.2 in wild-type salivary gland cells but decreased to 1.0 following *droj2* depletion (Figure 6G), indicating a marked reduction in the nuclear accumulation of EcRA. To further characterize this phenotype, we performed a time-course analysis of the EcRA N/C ratio after *droj2* knockdown. In wild-type salivary glands, the EcRA N/C ratio remained relatively constant, ranging from 2.34 to 2.74, whereas *droj2* depletion caused a progressive decline, reaching a minimum value of 0.89 approximately 2 h after knockdown (Figure 6H). To determine whether this reduction resulted from enhanced nuclear export, larvae were treated with Leptomycin B (LMB), a specific inhibitor of Exportin 1 (CRM1)-mediated nuclear export (Kudo et al., 1998). LMB treatment markedly restored the EcRA N/C ratio in *droj2*-depleted salivary glands from 1.4 to 2.1 (Figure 6I). These results indicate that inhibition of CRM1-dependent nuclear export partially rescues the loss of nuclear EcRA caused by *droj2* depletion.

**FIGURE 7:**
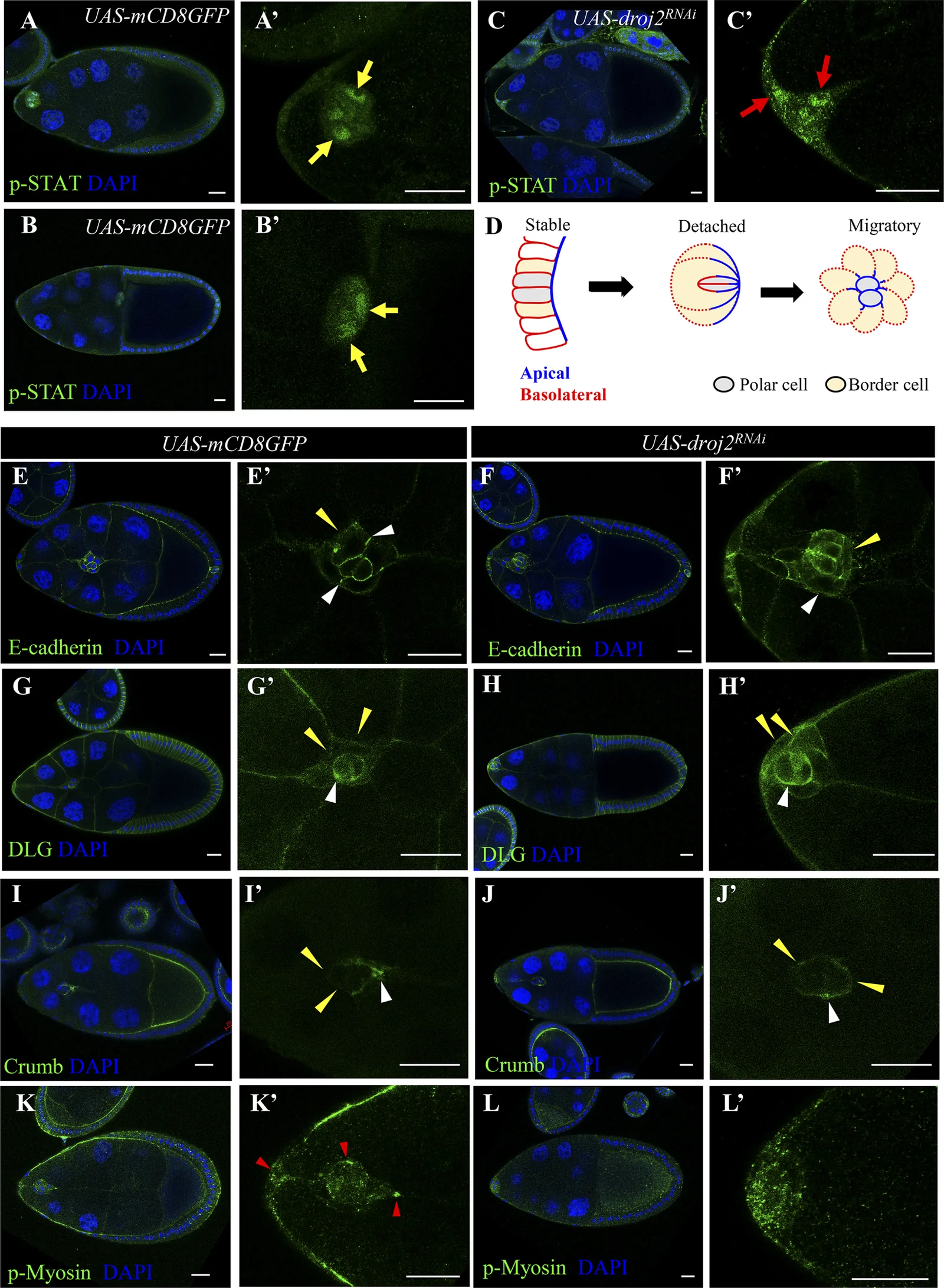
Knockdown of droj2 disrupts cell junctions, polarity, and Myosin contractility in border cells. **(A–C)** Representative confocal images of border cell clusters stained with anti-pSTAT (green) and DAPI (blue). **(D)** Schematic illustration of the distribution of polarity proteins in border cells. **(E–L)** Confocal images of border cell clusters stained for E-cadherin (green; **(E, E′, F,F′)**), Dlg (green; **(G, G′, H,H′)**), Crumbs (green; **(I, I′, J,J′)**), p-Myosin (green; **(K, K′, L,L′)**), and DAPI (blue) for the indicated genotypes. Scale bars, 20 μm.

Given the role of Droj2 in regulating EcRA nuclear localization, we next examined whether JAK/STAT signaling was also affected by monitoring nuclear phospho-STAT (pSTAT), a well-established readout of JAK/STAT pathway activation (Yan et al., 1996). During border cell migration, strong nuclear pSTAT staining was observed in border cells (Figures 7A,B’). Similarly, nuclear pSTAT localization was maintained in *droj2*-depleted border cells despite their failure to migrate beyond the anterior tip of the egg chamber (Figure 7C,C’).

To determine whether *droj2* depletion affects the redistribution of adhesion and polarity proteins during border cell migration, we examined the localization of E-cadherin, Crumbs (Crb), Discs large (Dlg), and phospho-Myosin (p-Myosin) (Figure 7D). In control border cell clusters, E-cadherin was enriched at the interfaces between border cells (white arrowheads, Figure 7E’) but was reduced at the cluster periphery facing the nurse cells (yellow arrowhead, Figure 7E’). Similarly, the apical marker Crb and the basolateral marker Dlg exhibited well-defined membrane localization (Tanentzapf et al., 2000; Miao et al., 2022) (Figure 7G’,I’). In contrast, *droj2* knockdown resulted in a less distinct distribution of E-cadherin, Crb, and Dlg within the border cell cluster (Figure 7F’,H’,J’). We further examined the distribution of p-Myosin, which was enriched in puncta at protrusive extensions and at the site of contact with anterior follicle cells during border cell detachment in control egg chambers (red arrowheads, Figure 7K’). Upon *droj2* depletion, p-Myosin staining exhibited a more uniform distribution throughout the border cell cluster (Figure 7L’). Together, these results show that depletion of Droj2 alters the distribution of E-cadherin, Crb, Dlg, and p-Myosin, indicating defects in the organization of adhesion, polarity, and contractile machinery during border cell migration.

## Discussion

Steroid hormone receptors are differentially regulated at the level of nuclear entry versus nuclear function. Whereas the glucocorticoid receptor undergoes ligand-dependent nuclear translocation, the estrogen receptor α is predominantly nuclear at steady state and is instead controlled by mechanisms governing receptor stability, chromatin engagement and transcriptional competence (Htun et al., 1999; Pratt and Toft, 2003; Echeverria and Picard, 2010). Our data indicates that the ecdysone receptor follows a similar paradigm during oogenesis, as EcRA accumulates in the nucleus but undergoes temporally regulated downregulation (Jang et al., 2009). Notably, EcR function depends on molecular chaperones, including Hsp90 and Hsc70, which are required for receptor maturation and activity (Arbeitman and Hogness, 2000). Consistent with this framework, Hsp70 and its Hsp40 co-chaperone *(Drosophila* DnaJ-1) are required for border cell migration, where perturbation of either component leads to migration defects and altered signaling outputs. Moreover, DnaJ-1 physically and genetically interacts with the guiding signal receptor PDGF/VEGF (PVR), indicating that Hsp40-Hsp70 complexes can regulate signaling pathways by modulating receptor function rather than simply promoting protein folding (Cobreros et al., 2008).

**FIGURE 8:**
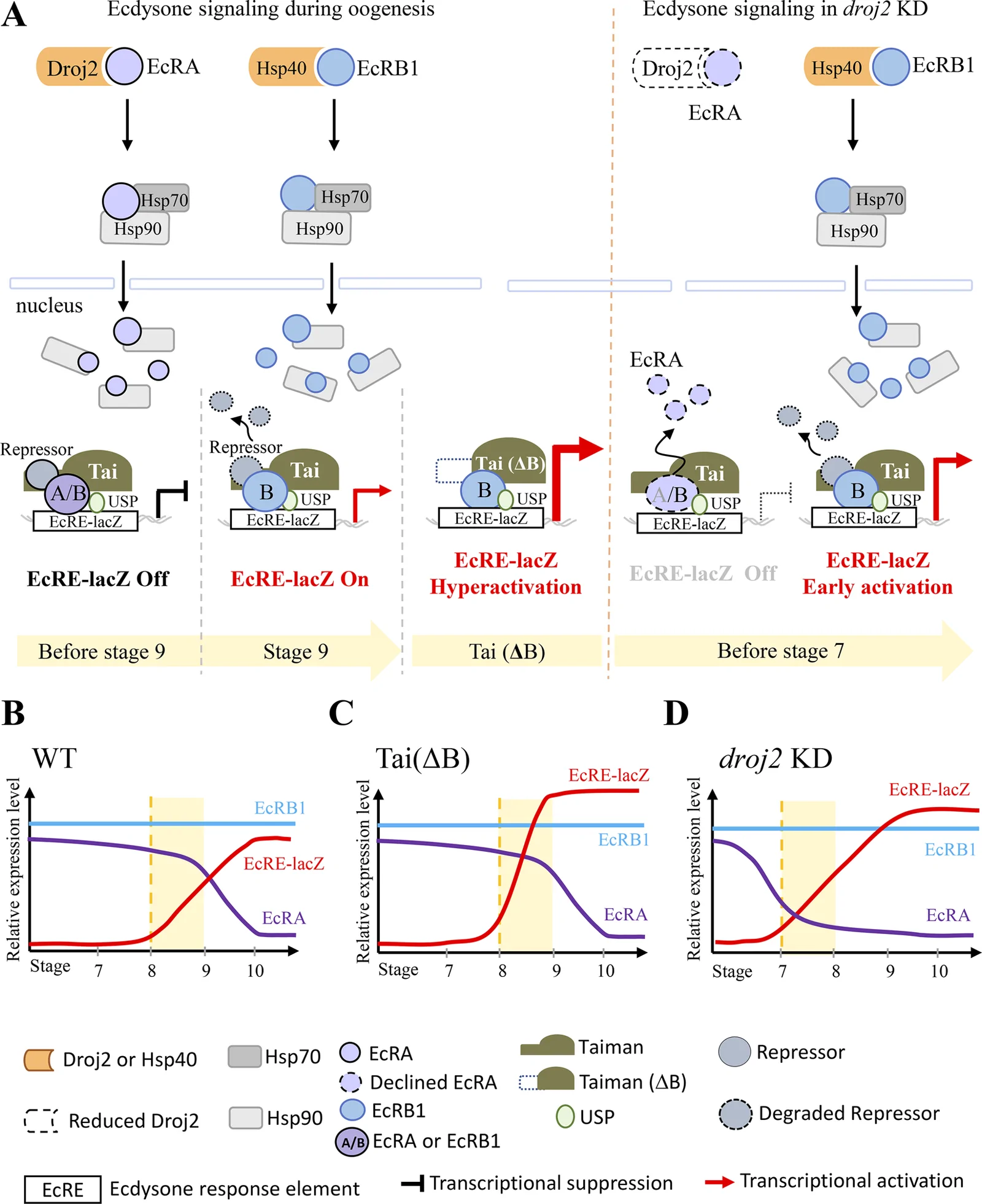
Proposed model of Droj2-mediated temporal regulation of EcRA dynamics and ecdysone signaling during border cell migration. **(A)** Proposed model illustrating the regulation of *EcRE-lacZ* activity by EcRA and EcRB1 in wild-type, Tai(ΔB)-expressing, and droj2 knockdown ovaries. **(B–D)** Schematic diagrams showing the relative expression levels of EcRA, EcRB1, and EcRE-lacZ in wild-type **(B)**, Tai(ΔB)-expressing **(C)**, and droj2 knockdown **(D)** egg chambers.

In this context, the premature loss of nuclear EcRA following *droj2* depletion is unlikely to result primarily from defective nuclear import but instead reflects a failure to maintain EcRA within the nucleus. Our kinetic analysis of the EcRA nuclear-to-cytoplasmic (N/C) ratio demonstrates a progressive reduction in nuclear EcRA following *droj2* knockdown, supporting a role for Droj2 in maintaining the nuclear retention of EcRA. Although we cannot completely exclude a contribution of impaired nuclear import, the ability of Leptomycin B (LMB), a well-established inhibitor of Exportin 1 (CRM1)-mediated nuclear export, to partially restore the EcRA N/C ratio provides strong evidence that defective nuclear retention is a major mechanism underlying the premature loss of nuclear EcRA. These findings suggest that Droj2 functions to preserve the temporal dynamics of EcRA, thereby preventing premature activation of ecdysone signaling during early oogenesis (Figures 8A,D). Together, these observations support a model in which Droj2 contributes to the timely decline of nuclear EcRA, thereby ensuring the gradual activation of ecdysone signaling (Figures 8A,B). Consistent with this model, both loss- and gain-of-function of Droj2 disrupt this coordination and impair border cell migration. Furthermore, our previous studies demonstrated that both reduced and excessive ecdysone signaling also disrupt border cell migration. Loss of *taiman* suppresses *EcRE-lacZ* activity, whereas expression of the hyperactive form of Tai, Tai(ΔB), escalates ecdysone signaling (Figures 8A,C), and both conditions lead to severe migration defects (Jang et al., 2009). We propose that Droj2, as an Hsp40 co-chaperone, acts through the Hsp70 machinery to preserve EcRA in a folding-competent and functionally active state, thereby preventing its premature turnover and sustaining its nuclear pool, consistent with the precocious activation of *EcRE-lacZ* observed upon *droj2* depletion. By conferring substrate specificity to Hsp70, Droj2 may selectively regulate EcRA stability, consistent with the established role of Hsp70-Hsp40 systems in controlling client protein stability and conformational dynamics (Kampinga and Craig, 2010; Rosenzweig et al., 2019; Wentink et al., 2026). Loss of Droj2 would therefore accelerate EcRA destabilization and nuclear depletion, resulting in precocious activation of ecdysone-responsive transcription.

These findings extend the functional scope of Hsp40 chaperones beyond their canonical roles in protein folding and quality control, positioning them as regulators of nuclear receptor dynamics and signaling timing. By coupling proteostasis to the stability and availability of a nuclear-resident hormone receptor, Droj2 provides a mechanism for fine-tuning the temporal window of steroid hormone responsiveness during development. More broadly, this work suggests that chaperone systems can act as upstream determinants of signaling thresholds, integrating protein homeostasis with transcriptional control (Mayer and Bukau, 2005; Rosenzweig et al., 2019). Given the conservation of Hsp70-Hsp40 machinery and steroid receptor regulation across species, similar mechanisms may operate in other hormone-responsive contexts, where modulation of nuclear receptor stability, rather than localization, serves as a key regulatory node (Grad and Picard, 2007).

## Materials and methods

### *Drosophila* strains and genetics

The following *Drosophila* strains were used in this study: *w1* ^*1118*^ and *UAS-mCD8GFP* served as controls. For the modifier screen, *slboGAL4* was used to drive transgene expression in border cells (Rørth et al., 1998). To prevent developmental lethality associated with the *tubGAL80* ^*t*^ background, flies were maintained at 31 °C for 16 h before dissection. *UAS-tai(ΔB)23A4* was used to induce hyperactivation of ecdysone signaling.

The chromosome II deficiency kit (Df (2)ED), including Df (2L)ED and Df (2R)ED lines spanning chromosome II, was obtained from the *Drosophila* Genetic Resource Center (DGRC) and the Bloomington *Drosophila* Stock Center (BDSC). Candidate genes identified from the deficiency screen were further validated using available mutant alleles and RNAi lines obtained from BDSC, the Vienna *Drosophila* Resource Center (VDRC), and DGRC. These included *droj2*, *l(2)gl*, *spin*, and additional candidates listed in Supplementary Table S1.

The following lines were used for *droj2* analysis: *droj2* ^*GS5242*^ (DGRC20809), *droj2* ^*GS21515*^ (DGRC202858), *UAS-droj2* ^*RNAi*^ *-1* (VDRC104880), *UAS-droj2* ^*RNAi-*^ *2* (VDRC 23637), were acquired from the Kyoto Stock Center (DGRC) and the Vienna *Drosophila* Resource Center (VDRC). Additional strains obtained from BDSC included: *tub-GAL80* ^*t*^ (BDSC 7019), *FRT82B* (BDSC 5748), *FRT82B*, *ubi-mRFP* (BDCS 30555) and *24BGAL4* (BDSC1761).

To generate mitotic clones, females carrying *hsFLP; FRT82B, ubi-mRFP/FRT82B*, *droj2* ^*GS5242*^ were heat shocked at 37 °C for 1 h twice daily for three consecutive days. Flies were subsequently maintained at 25 °C for 5 days prior to ovary dissection and immunostaining. For temporal induction using the *c306GAL4, tubGAL80* ^*t*^ system, flies were cultured at 18 °C and shifted to 31 °C for 2 days and fed sequentially with dry and wet yeast before ovary dissection. For salivary gland experiments, transgene expression was induced using *24BGAL4*. Flies were maintained at 25 °C and shifted to 29 °C for 1–2 days before dissection, and wandering third-instar larvae were selected for salivary gland analysis.

### Generation of *UAS-droj2* transgenic flies

To generate *UAS-droj2* transgenic flies, the genomic sequence of *droj2* was amplified from wild-type *Drosophila* melanogaster genomic DNA by PCR. Primers were designed to introduce restriction enzyme sites for subcloning into the pUASp vector. The forward primer contained a KpnI site (5′-GGT​ACC​ACA​CTG​GTT​TCC​AGT​TTT​CTT​C-3′), and the reverse primer contained a SacII site (5′-CCG​CGG​AAG​AAT​ATA​CAG​CAT​TTG​GGT​TG-3′). The resulting 3.2-kb PCR fragment was digested with KpnI and SacII and ligated into the pUASp vector linearized with the same enzymes. The pUASp-*droj2* construct was verified by DNA sequencing and introduced into *Drosophila* embryos by microinjection. Stable transgenic lines carrying the *UAS-droj2* transgene were subsequently established for further analysis.

### Immunohistochemistry

Adult female flies were fed wet yeast paste before dissection to promote ovary development. Salivary glands were dissected from wandering third-instar larvae. Ovaries and salivary glands were dissected in Grace’s Insect Medium (Sigma-Aldrich, G8142) and fixed on ice for 30 min in 1× phosphate-buffered saline (PBS) containing 4% paraformaldehyde and 1 mM CaCl_2_. Samples were washed three times with PBS containing 0.3% Triton X-100 (PBT) and incubated in PBT overnight at 4 °C to improve tissue permeability. Primary antibodies were incubated at 4 °C for 12–18 h, followed by three washes in PBT and incubation with Alexa Fluor-conjugated secondary antibodies (Invitrogen; 1:200) for 1 h at room temperature. The following primary antibodies obtained from the Developmental Studies Hybridoma Bank (DSHB) were used: rat anti-E-cadherin (DCAD2, 1:20), mouse anti-Dlg (4F3, 1:10), mouse anti-Crb (Cq4, 1:10), mouse anti-Armadillo (N27A1, 1:10), mouse anti-*β-galactosidase* (40-1a, 1:20), mouse anti-EcR-A (15G1a, 1:20), and mouse anti-EcR-B1 (AD4.4, 1:10). Rabbit anti-Taiman (1:10,000) was generously provided by Denise J. Montell. For nuclear and actin staining, DAPI was used at 1:10,000 and Phalloidin (Sigma P1951 and P5282) at 1:5,000. Rabbit anti-p-Myosin (phospho-Myosin Light Chain 2 (Ser19), Cell Signaling, 3,671) was used at 1:20. For phospho-Stat (p-Stat) immunostaining, ovaries were dissected in S2 medium supplemented with 10% fetal bovine serum (FBS) and 1× phosphatase inhibitors (PhosSTOP, Sigma-Aldrich), followed by fixation in 4% paraformaldehyde in 1× PBS containing 1× phosphatase inhibitors for 2 h at 4 °C. Samples were washed three times with 0.5% PBT (PBS containing 0.5% Triton X-100) and incubated overnight at 4 °C with anti-p-Stat antibody (1:400; Gift from Dr. Yu-Chen Tsai, Tunghai University) diluted in 0.3% PBT. Details were as described in previous research (Wu et al., 2022). For Crumbs immunostaining, ovaries were fixed for 10 min at room temperature, treated with methanol for 5 min, washed with PBT (3 × 40 min), and then incubated with the primary antibody (Tanentzapf et al., 2000).

Samples were mounted in ibidi mounting medium (Cat. No. 50001) and stored at 4 °C. Images were acquired using a Carl Zeiss LSM780 confocal microscope at the Instrument Development Center of National Cheng Kung University and processed using ZEN 2010 and Adobe Photoshop software.

### Kinetic assay of EcRA nuclear transport in salivary glands

For EcRA nuclear transport assays, flies were maintained at 18 °C to minimize transgene expression during development. Third-instar larvae were shifted to 29 °C for 0, 1, 2, or 4 h to induce transgene expression using the 24BGAL4/UAS system before salivary glands were dissected for immunostaining. For leptomycin B (LMB) treatment, third-instar larvae were transferred to yeast paste containing 10 μM LMB and incubated at 29 °C for 2 h before salivary gland dissection. Control larvae were treated with an equivalent volume of ethanol (vehicle) under the same conditions.

### Quantitative analysis of confocal images

Egg chambers from stages 7–10 were identified according to morphology and the anterior-posterior/dorsal-ventral (AP/DV) ratio. For *EcRE-lacZ* reporter analysis, fluorescence intensity was quantified from anti-β-galactosidase staining using Fiji software. Regions of interest (ROIs) were manually defined based on F-actin staining. In stage 7–8 egg chambers, ROIs were drawn around anterior follicle cells, whereas in stage 9–10 egg chambers, ROIs were drawn around border cells. Corresponding nurse cell regions within the same egg chamber were used as internal controls. Relative *EcRE-lacZ* activity was calculated as the fluorescence intensity ratio between border cells (or anterior follicle cells) and nurse cells.

For quantification of EcR-A and EcR-B1 expression in egg chambers and salivary glands, ROIs were manually drawn around individual nuclei using DAPI staining to define nuclear boundaries. In egg chambers, anterior follicle cell nuclei were analyzed at stages 7–8, whereas border cell nuclei were analyzed at stages 9–10. For salivary gland analysis, individual nuclei were quantified independently, with multiple nuclei analyzed per gland. Mean fluorescence intensity was measured from the EcR-A or EcR-B1 channel. Background fluorescence was measured from adjacent regions lacking specific signal within the same focal plane and subtracted from the nuclear fluorescence intensity. Corrected fluorescence intensity was calculated as:

*I*_*corrected*_ = *I*_*nuclear*_ *-I*_*background*_. Corrected fluorescence values were used for subsequent statistical analyses and are presented in arbitrary units (a.u.). Data are shown as mean ± SD. Similar approach was applied for nuclear-to-cytoplasmic (N/C) ratio analysis of EcR-A in salivary glands, nuclear ROIs were manually defined using DAPI staining, whereas whole-cell ROIs were delineated using phalloidin staining. Cytoplasmic ROIs were generated by subtracting the nuclear ROI from the corresponding whole-cell ROI. The N/C ratio of EcR-A was calculated as (*I*_*nucleus*_ *− I*_*background*_ *)/(I*_*cytoplasm*_ *− I*_*background*_). All quantitative data are presented as the mean ± SD. Statistical significance was determined using an unpaired, two-tailed Student’s t-test. n. s., not significant; *P < 0.05; **P < 0.01; ***P < 0.001.

## Data Availability

The raw data supporting the conclusions of this article will be made available by the authors, without undue reservation.
