# Supplementary material for: Hsp40 co-chaperone Droj2 temporally regulates EcRA dynamics during collective cell migration
Source: Front Cell Dev Biol. 2026 Aug 18;14:1881376. doi: 10.3389/fcell.2026.1881376 (PMC13529688; doi:10.3389/fcell.2026.1881376)
Supplement: Supplementary file 1 [file Image1.pdf]

Supplemental Figure 1

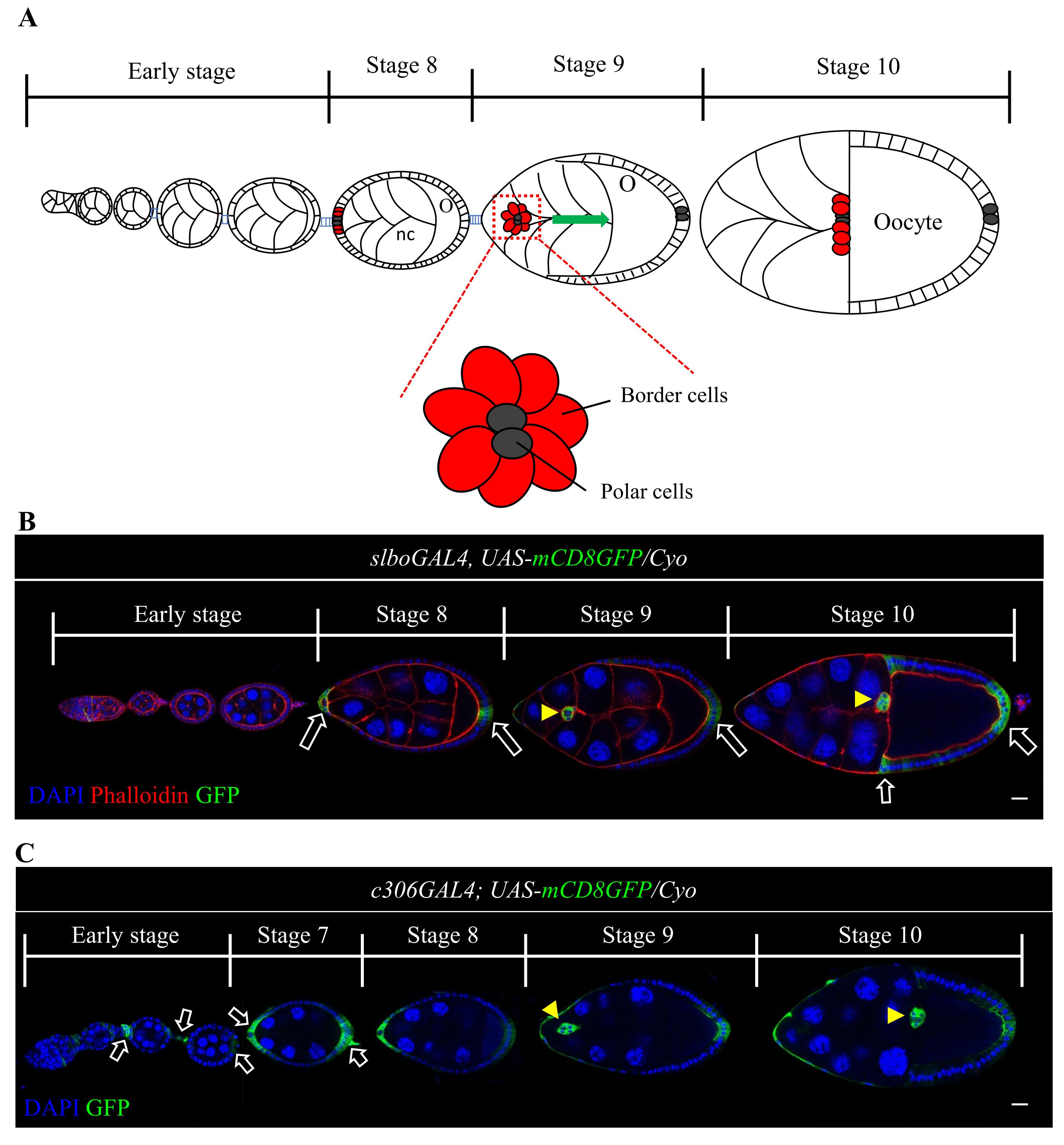

Figure S1. Schematic representation of border cell migration in *Drosophila* oogenesis.
(A) The *Drosophila* ovary contains ~16 ovarioles, each comprising a series of developing egg chambers. During early oogenesis, one germline cell differentiates into the oocyte (O), while the other 15 ones become nurse cells (nc). Egg chambers are separated by stalk cells (blue) and two pairs of polar cells (dark gray) at their anterior and posterior ends. At stage 9, 6 to 8 follicle cells are specified as border cells (red) and recruited by the anterior polar cells to form a migratory cluster. This cluster detaches from the anterior epithelium and migrates toward the oocyte, reaching its destination by stage 10. Representative confocal images of egg chambers during oogenesis. GFP marks the *slboGAL4* (B) or *c306GAL4* (C) expression domain, presenting in border cells (yellow arrowheads) and subsets of anterior and posterior follicle cells (white open arrows). Nuclei are labeled with DAPI (blue), and F-actin is visualized by phalloidin staining (red) to outline cell morphology.

Supplemental Figure 2

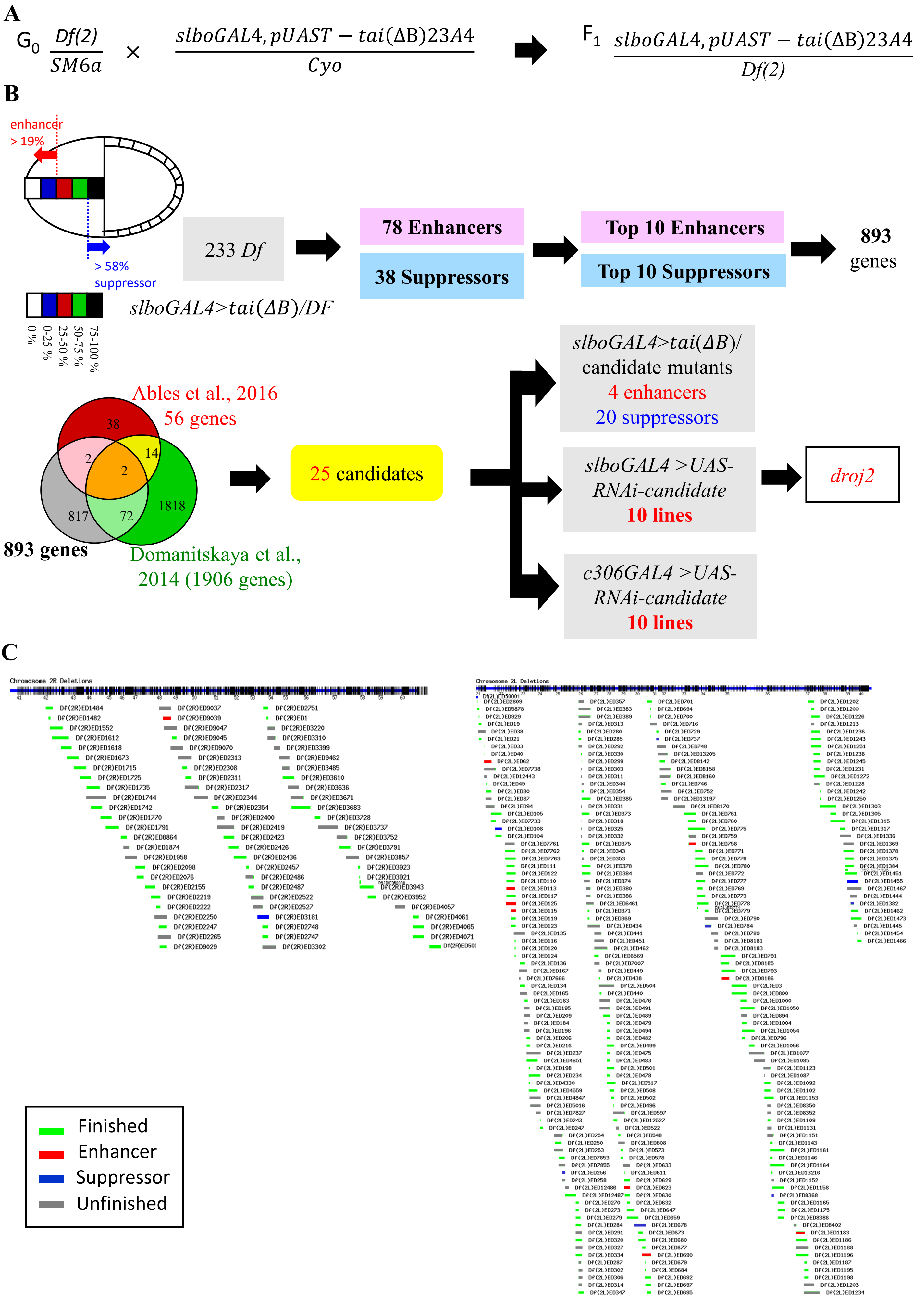

Figure S2. Genetic screen identifies novel loci modifying border cell migration defects caused by Tai(ΔB).

(A) Schematic overview of the genetic screen using deficiency lines to identify modifiers of Tai(ΔB)-induced border cell migration defects. (B) The schematic of screening criteria used to define deficiency lines as enhancers or suppressors of *UAS-tai(ΔB)*-mediated migration defects. A total of 893 genes within the top 10 enhancer and suppressor deficiency intervals were cross-referenced with published gene sets associated with ecdysone signaling, which retrieves 25 candidate genes. These candidates were subsequently re-examined for their genetic interaction with *UAS-tai(ΔB)*-induced migration defects and further validated by RNAi knockdown using either *slboGAL4* or *c306GAL4* to assess their roles in border cell migration. (C) Cytological map of second chromosome deficiencies from the DrosDel deficiency kit. Cytological positions are based on FlyBase annotations. Deficiency lines (green) were tested for genetic interaction with *slboGAL4 >UAS-tai(ΔB)23A4*. Among these, the top 10 enhancers are highlighted in red, and the top 10 suppressors are indicated in blue. Gray regions represent deficiency lines that were not analyzed.

Supplemental Figure 3

A

Rank	Gene (mutant or RNAi line)	75-100 %	n
1	*l(2)gl*^{4}	99	74
2	*spin*^{11F5}	98	92
3	*spin*^{Delta58}	96	24
4	*trs23*^{HMS01765}	95	117
5	*Sec24CD*^{1}	94	83
6	*hr39*^{C105}	92	100
7	*tor*^{2L}	89	85
8	*hr39*^{Scim}	81	83
9	*tor*^{E16K}	79	42
10	*dbr*^{EP9}	74	94
11	*socs36E*^{EY06665}	74	31
12	*unc-104*^{d11204}	74	103
13	*mdy*^{QX25}	73	74
14	*tor*$^{CB2-1}$	69	107
15	*lis-1*^{D}	67	139
16	*rilpl*^{JF02940}	65	108
17	*mdy*^{GD1749}	62	110
18	*gcm*^{KG01117}	62	68
19	*nLaz*^{f03602}	62	112
20	*hoip*^{k07104}	57	116
	slboGAL4>tai(ΔB)	**46**	**5578**
4	*trx-2*^{BG02804}	28	148
3	*CG31886*^{HMS03030}	28	120
2	*iris*^{GLC01709}	25	64
1	*droj2*^{GS5242}	5	238

B

Rank	Gene (mutant or RNAi line)	75-100 %	n
	slboGAL4>lacZ	**90**	**503**
10	*rilpl*^{GD7304}	76	45
9	*CG31886*^{GD1020}	76	153
8	*iris*^{GLC01709}	76	181
7	*droj2*^{KK107834}	75	528
6	*Sec24CD*^{GD4052}	75	161
5	*gcm*^{GD1452}	75	599
4	*spin*^{JF02782}	67	290
3	*droj2*^{GD14050}	65	221
2	*dbr*^{GD7281}	45	31
1	*dimm*^{GD4377}	35	470

C

Rank	Gene (mutant or RNAi line)	75-100 %	n
	c306GAL4>lacZ; tubGAL80^{ts}	97	123
10	*droj2*^{GD14050}	49	275
9	*spin*^{JF02782}	47	233
8	*sec24AB*^{KK101569}	44	114
7	*acer*^{GD1187}	36	14
6	*lis-1*^{GD1480}	35	66
5	*droj2*^{KK107834}	35	145
4	*dimm*^{GD4377}	34	32
3	*l(2)gl*^{KK100777}	32	41
2	*dimm*^{KK100204}	23	72
1	*nLaz*^{KK107553}	18	86

Figure S3. Prioritization of candidate genes from a deficiency-based modifier screen of Tai(ΔB)-induced migration defects.

Based on the integrative analysis shown in Figure 2B, 25 candidate genes were selected for functional validation in border cell migration. RNAi or mutant lines were crossed with *slboGAL4, tai(ΔB)/CyO* (A), *slboGAL4* (B), or *c306GAL4* (C) to evaluate their roles in border cell migration. The table summarizes the results of the secondary screen. *n* indicates the number of stage 10 egg chambers analyzed for each genotype.

Supplemental Figure 4

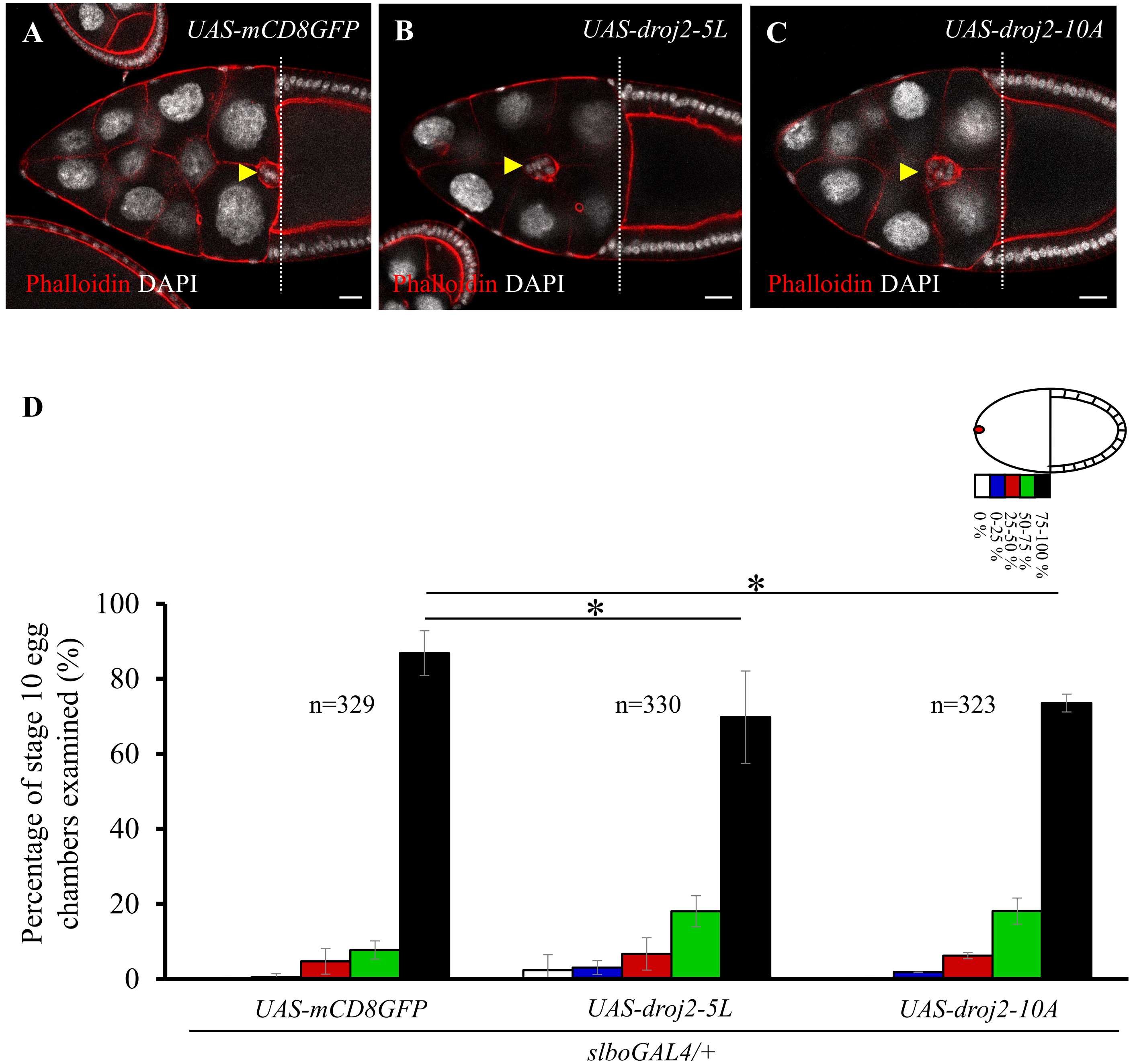

Figure S4. Droj2 overexpression impairs border cell movement.
(A-C) Representative confocal images of stage 10 egg chambers showing border cell migration defects in the indicated genotypes. Border cell clusters are marked by yellow arrowheads. Nuclei are labeled with DAPI (white), the oocyte border is outlined by white dashed lines, and F-actin is visualized by phalloidin staining (red) to reveal egg chamber morphology. (A) GFP (green) marks the *slboGAL4* expression domain and serves as the control. (B, C) Overexpression of two independent *UAS-droj2* insertion lines impaired border cell migration. (D) Quantification of border cell migration in the indicated genotypes. n represents the number of stage 10 egg chambers analyzed. Data are presented as mean ± SD. Statistical significance was determined using an unpaired two-tailed Student's t-test. (*$P < 0.05$). Scale bars, 20 µm.

Supplemental Figure 5

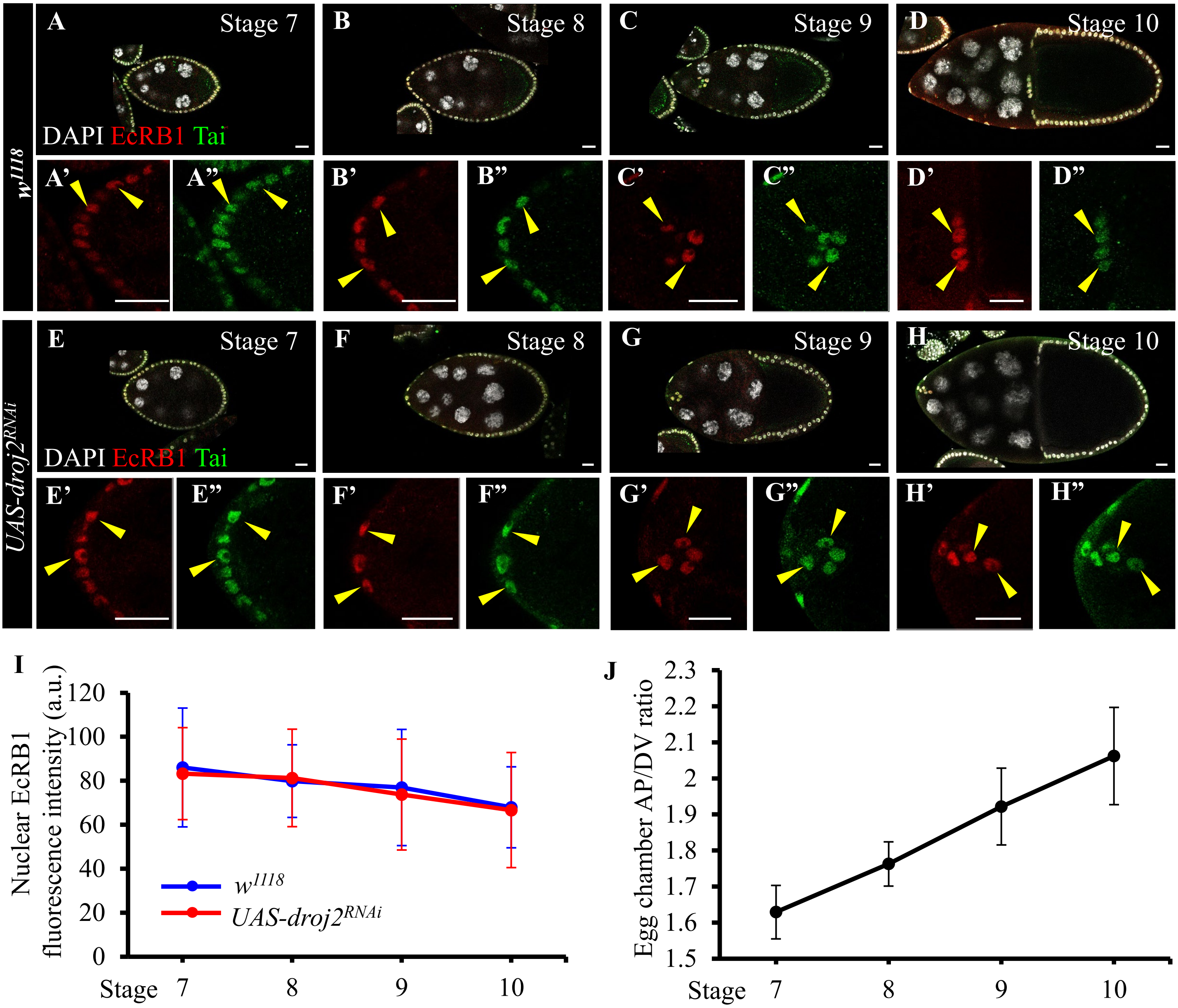

Figure S5. Suppression of *droj2* does not affect EcRB1 or Taiman expression

UAS-droj2^{RNAi} is activated in early oogenesis by *c306GAL4*. DAPI (white), anti-EcRB1 (red), and anti-Tai (green) staining were used to label follicle cell nuclei in the indicated genotypes (A-H). The expression levels of EcRB1 and Taiman were comparable between wild type,*w*^{1118}, and *droj2* RNAi conditions. Yellow arrowheads mark anterior follicle cells (A', A", B', B", E', E", F', F") or border cells (C', C", D', D", G', G", H', H"). (I) Quantification of EcRB1 expression was performed in 328 anterior follicle cells across the indicated genotypes. (J) Egg chamber stages were defined based on the AP/DV ratio, with measurements obtained from 155 egg chambers. Scale bars, 20 µm.
